# Mastodon over Mammon: towards publicly owned scholarly knowledge

**DOI:** 10.1098/rsos.230207

**Published:** 2023-07-19

**Authors:** Björn Brembs, Adrian Lenardic, Peter Murray-Rust, Leslie Chan, Dasapta Erwin Irawan

**Affiliations:** ^1^ Institut für Zoologie - Neurogenetik, University of Regensburg, Regensburg 93040, Germany; ^2^ Wiess School of Natural Sciences Ringgold Standard Institution - Earth Science, Rice University, Houston, Texas 77005, USA; ^3^ Department of Chemistry, University of Cambridge, Cambridge, CB2 1EW UK; ^4^ Global Development, University of Toronto, Toronto Scarborough, Ontario M1C 1A4 Canada; ^5^ Department of Earth Science, Institut Teknologi Bandung, Bandung, 40132 Indonesia

**Keywords:** Mastodon, Mammon, scholarly, knowledge, Twitter, Fediverse

## Abstract

Twitter is in turmoil and the scholarly community on the platform is once again starting to migrate. As with the early internet, scholarly organizations are at the forefront of developing and implementing a decentralized alternative to Twitter, Mastodon. Both historically and conceptually, this is not a new situation for the scholarly community. Historically, scholars were forced to leave social media platform FriendFeed after it was bought by Facebook in 2006. Conceptually, the problems associated with public scholarly discourse subjected to the whims of corporate owners are not unlike those of scholarly journals owned by monopolistic corporations: in both cases the perils associated with a public good in private hands are palpable. For both short form (Twitter/Mastodon) and longer form (journals) scholarly discourse, decentralized solutions exist, some of which are already enjoying some institutional support. Here we argue that scholarly organizations, in particular learned societies, are now facing a golden opportunity to rethink their hesitations towards such alternatives and support the migration of the scholarly community from Twitter to Mastodon by hosting Mastodon instances. Demonstrating that the scholarly community is capable of creating a truly public square for scholarly discourse, impervious to private takeover, might renew confidence and inspire the community to focus on analogous solutions for the remaining scholarly record—encompassing text, data and code—to safeguard all publicly owned scholarly knowledge.

**Figure 1.**
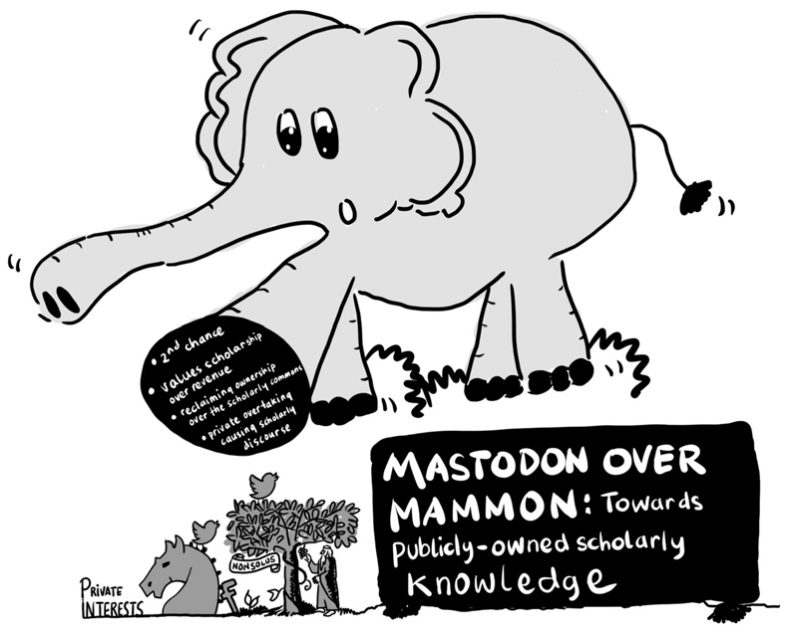
Image credit: DEI.

## 1. A public good in private hands—again

With the turmoil surrounding Elon Musk's handling of his Twitter take-over, the problems associated with a public good in private hands have again become a focus of public attention. For scientists, the situation is not unlike that of 2009, when a social media platform widely used by scholars, FriendFeed, was bought by Facebook and subsequently shut down [1]. This instance was only one of several where the dangers of private, profit-oriented organizations owning platforms used for scholarly discourse became palpable for everyone involved and were widely discussed. One of the outcomes of these discussions over the last 15 years is a set of open standards for social technologies that mimic the open standards underlying the wider internet and web, the World Wide Web Consortium's ActivityPub [2]. In 2009, scholars started to leave FriendFeed and migrate to Twitter, founding what has grown to a community of about half a million researchers and is often referred to as #ScienceTwitter [3]. Now, much of #ScienceTwitter is migrating to Mastodon [4], an application based on ActivityPub in what is called the ‘Fediverse’ [2]. Analogous to web or email servers, Mastodon runs on so-called instances (servers) and while anybody can implement such instances, nobody can control all of them, just like nobody controls all email or web servers [5]. While corporate capture is a risk even for such decentralized technologies (see for example GMail or Meta's ‘Threads’), decentralization provides means for defending against corporate capture. See our companion publication [6] for more safeguards against corporate capture. We identify parallels between private ownership of #ScienceTwitter and private ownership of scholarly journals, prompting a proposal to safeguard the entire scholarly record from corporate vagaries.

## 2. A golden opportunity

Even before Mr Musk bought Twitter, especially at-risk scholars of various minority groups were already leaving the increasingly toxic site and founded scholar.social on Mastodon [7]. Now, the first scholarly organizations are supporting the Fediverse: the international Neuromatch (neuromatch.social), the European Laboratory for Learning and Intelligent Systems (ellis.social), the Dutch Centre for Science and Technology Studies (social.cwts.nl), the Irish Dublin Institute for Advanced Studies (mastodon.dias.ie) or the German Helmholtz Centers (helmholtz.social), Max Planck Society (social.mpdl.mpg.de) or Society for Digital Humanities (fedihum.org) have already implemented their own Mastodon instances. Even single individuals are stepping up and providing instances for their communities (for instance, Giorgio Gilestro is hosting drosophila.social). In the Netherlands, SURF, the collaborative organization for information technology in Dutch education and research, have started a pilot to explore how a Mastodon environment for education and research in the Netherlands can take shape [8]. We call on more scholarly organizations to host their own Mastodon instances or join collaborative projects in the Fediverse (see also [9–12]). We believe there are several good reasons why scholarly societies, in addition to public institutions such as universities and research institutes, are particularly well-placed to take advantage of this golden opportunity. With ‘scholarly societies’ we here refer to scholarly organizations that exist to promote an academic discipline, profession, or a group of related disciplines. Many scholarly or learned societies are professional associations [13].

## 3. Striking parallels

Twitter is not the only case where scholars are struggling with a public good in private hands. In scholarly publishing, scientists and the wider public are similarly exposed to the whims of a few, large corporations. It is worth remembering that a key rationale of the Open Access (OA) movement was to reclaim the public commons and to allow scholars themselves to be in charge of the governance of knowledge production and circulation. The open repository movement was very much built on the idea of what one now calls the Fediverse. It has taken another 20 years for the preprint movement to take hold beyond some mathematics/physics fields, and for repository developers to create tools and services that serve scholarship better than journals. For instance, from these developments arose CORE as the world's largest aggregator of open access research papers from repositories and journals. Above and beyond repositories, such ‘decentralized’ solutions are being discussed more and more as the most promising solutions for a whole host of pressing infrastructure problems (e.g. [14,15]).

Given the speed at which digital technology evolves, why have these academic developments taken decades to materialize? There are several answers to this question, but different actions of learned societies during these decades deserve to be highlighted. The following examples also serve to highlight the thought processes in different academic and geographical areas and together with the parallels between journal publishing and social technologies instruct our conclusion that academia may profit from learning from past mistakes.

## 4. Professionalization of some scholarly societies

Scholarly societies have been the bedrock of organized scholarship for centuries. Then, as now, scholars were rarely motivated by fortune or fame, but commonly by curiosity and a fundamental idealism to further humanity and knowledge. For the privileged men founding the first society in 1660, ‘Their first purpose was no more, then onely the satisfaction of breathing a freer air, and of conversing in quiet one with another, without being ingag'd in the passions, and madness of that dismal Age’ [16, p. 54]. Later, societies provided circulation and support for an expensive, difficult and sometimes risky passion.

Today, societies organize meetings of tens of thousands of professional researchers, publish journals, award prizes, promote early career researchers, lobby politicians, initiate and maintain public outreach efforts and provide expertise as a public service. Such large organizations require funding and professional staff. From their public records, we learn that, for instance, the top 10 staff of the American Psychological Association (APA) receive more than US$ 4 million in compensation annually. Very similar figures were reported by the Massachusetts Medical Society, the publisher of the *New England Journal of Medicine*. ‘Management and governance’ are the largest expense also for the American Anthropological Association (AAA) with 29% of their uses of their annual revenue. The American Association for the Advancement of Science (AAAS), the society that publishes *Science Magazine, also* pays their executives more than US$ 4 million every year.

While the societies above were chosen arbitrarily, their sources of revenue are fairly similar in that membership dues only make up between 2 and 28%, while publishing income ranges from 28 to 88% of their annual budget. It is easy to find other societies with analogous numbers (table 1).

**Table 1.** Rounded figures for arbitrarily selected scholarly societies. (Data were sourced from, e.g. forms 990 (publicly available for US-based societies), or from the society's financial reports on their websites (other countries) from one of the last 5 years.)

	revenue	publishing		membership	
	US$M	US$M	% of rev.	US$M	% of rev.
American Anthropological Association	5.3	1.5	28	1.5	28
American Association for the Advancement of Science	114	62	54	9	8
American Chemical Society	670	558	83	18	3
American Economic Association	11	5.1	46	0.6	6
American Geophysical Union	42	18	43	2	5
American Psychological Association	130	115	88	3.6	3
Biochemical Society	5.8	5.3	91	0.27	5
European Society for Evolutionary Biology	0.35	0.3	86	0.01	3
Federation of American Societies for Experimental Biology	7.2	2.9	40	0.9	13
Massachusetts Medical Society	131	103	78	2.4	2
Royal Society of Chemistry	75	64	85	4.8	6
Society for Neuroscience	26	7	27	4	14

The growth and professionalization of scholarly societies comes not only with advantages, but also with challenges and unintended consequences. For instance, the dependence on publishing revenue to fund professional staff comes with conflicts of interest for these employees in that their livelihoods depend on this revenue. Also for leading members and decision-makers of such a society, scholarship may drop in priority when the many programmes and benefits that members have become used to, also become dependent largely on a dominant source of income. With many societies outsourcing their publication business to one of the aforementioned large corporations, they risk becoming trapped in the middle between corporate and scholarly interests. The last 25 years provide ample documentation of how some societies have embraced an increasingly distributed networked scholarly community with diverse revenue streams, while others have had a harder time adapting.

## 5. Some societies lead by example.....

The landscape of scholarly societies is highly heterogeneous, both within and between fields. Thus, it is not difficult to find examples where learned societies embrace new technologies to empower their members and further their mission and purpose.

In the Global North, perhaps the most recognized effort of scholarly societies in social media is the Humanities Commons (HC). The network enables scholars, researchers, practitioners, teachers and students to create a professional profile, discuss common interests, develop new publications and share their work. It is free to use and funded by grants and voluntary contributions. Modelling on the Fediverse, the HC is built upon a cooperation of scholarly societies, investing in a shared infrastructure. It was started by the Modern Language Association (MLA) which launched MLA Commons in 2013. The close temporal proximity to the development of other social media is not coincidental: HC grew out of the research of humanities scholars studying communication networks in the early 2000s [17]. Of course, HC sports a Mastodon instance of its own, hcommons.social.

In the Global South, cooperation between scholarly communities for shared digital infrastructure has a long history (these initiatives are perhaps not led by scholarly ‘societies’ in the historical sense, but scholar-led nonetheless). Cooperative publishing organization SciELO was founded in 1997 and is now supporting 16 countries and provides open access to their scholarly publications. SciELO was initially funded by the São Paulo Research Foundation with support from the Latin American and Caribbean Center on Health Sciences Information. The founding director of SciELO was biochemist Rogerio Meneghini. Most if not all of the initial journals that joined SciELO were society-based. Thus, while not strictly run by scholarly societies, SciELO is an example where scholarly societies cooperate with funders and scholars, taking advantage of modern technologies to advance scholarship without a profit motive.

Another prominent cooperative endeavour of the Global South is Redalyc/AmeliCa. This is a cooperative infrastructure for scientific communication governed by an inter-institutional academy on a broad scale, with funding from diverse sources [18]. Also this organization is a product of cooperation between different classes of stakeholders, not just scholarly societies. Similar scholar-led and non-profit atmosphere in academic journal operation has been operating for decades in Indonesia, as another example [19]. Scholar-led academic activities in Indonesia are typically supported by both state-owned institutions and private universities. These activities encompass a wide range of endeavours, including research initiatives and the maintenance of academic journals. Global South scholars and their societies provide pro bono work and expertise to realize the largest open access network on the planet, despite encroachment by increasing performance assessment based on journal prestige.

Scholars and their learned societies embracing digital technologies are not restricted to the humanities or the Global South. Some science societies in rich countries are also spearheading the modernization of scholarly communication using cooperative approaches. The Spanish Society for Experimental Psychology supported public access to their research early on. Their flagship journal ‘Psicológica’ had been online only and open access as early as 1998. In 2022, the society took the journal from De Gruyter Open and started to publish all journal contents, including articles, peer-reviews, data and software code exclusively at DIGITAL.CSIC, the institutional open access repository of the Spanish National Research Council, at no cost for authors or readers. After an initial screening, all submitted manuscripts are uploaded as preprints. The open and transparent peer-review process entails that reviewers are required to disclose their identity and that the full text of their reviews also becomes publicly available.

These examples demonstrate that a community which realizes the value of a communal resource is willing to find creative ways to curate such shared commons. Quality control, constructive discourse, error-correction and constant improvements are inherently weaved into the fabric of scholarship. These initiatives remind us that funders should be more creative with their support. HC, SciELO, AmeliCA or Psicológica, based on social technologies and cooperation, provide a huge and growing value for their communities, at a fraction of the cost of the antiquated and often dysfunctional privately owned journals—and without charging authors or readers anything. Like, for example, Wikipedia more generally, the examples above show that high-quality, high-value scholarship in the digital age does not require huge funds and massive inequities, only dedicated communities, shared digital infrastructure and community governance.

## 6. ......while others are more hesitant

Not all societies chose cooperative infrastructures over corporate platforms. For many large scientific societies, over-reliance on publication funds for their programmes have prevented them from implementing other options that better serve their members and their missions.

One of the earliest initiatives to wrestle digital control over the means of scholarly discourse from publishing corporations was Harold Varmus' proposal for public access to the biomedical literature, dubbed eBioMed, in 1999. A large scholarly society, the Federation of American Associations for Experimental Biology and other societies openly opposed the plan [20], eBioMed was stripped down radically and is now known as PubMed Central [21]. Not much later, the AAA axed their highly progressive publishing project ‘AnthroSource’ [22] citing financial concerns and signed with publishing corporation Wiley instead. Around the same time, the APA also started to publicly oppose taking advantage of digital means to spread scholarly knowledge further [23]. When the National Institute of Health sought to overcome this reluctance by mandating public access in 2008, the American Chemical Society (ACS) raised legal concerns [24]. The ACS was soon joined by the Association of Learned and Professional Society Publishers (ALPSP), the international trade association representing society publishers, in opposing access to scholarly literature. By voicing concerns not only to the National Institutes of Health access mandate, but also to United Kingdom public access policies and plans by the Obama administration in the United States (US) in 2012 [25,26], the ALPSP has demonstrated consistent opposition to scholarly knowledge being widely disseminated. The ACS also sued shadow library Sci-Hub in an attempt to protect their revenue and traditional infrastructure. So concerned were some societies about their income that a mere modernization rumour triggered more than 100 of them to team up with commercial publishers in 2019 and write a pleading letter to then-US-president Trump, fearing ‘some scientific societies [may be] forced to close their doors' [27, p. 1]. In 2022, after decades of resistance, the AAAS allowed their authors to freely share their publications immediately upon publication [28]. These examples indicate that scholarly societies heavily reliant on publishing revenue are strongly influenced by the oligarch commercial publishing industry. This industry possesses substantial power to dictate research policies and publishing standards, similar to other influential sectors (cartels really) such as big pharma and big tobacco, notorious for exerting corporate control over scientific policy and research priorities [29]. Their influence extends to fashioning research evaluation metrics to align with their corporate interests [30]. One such example is Clarivate, a private equity firm that owns the ‘Web of Science’ and the ‘Journal Citation Reports', which serve as the authorities on Journal Impact Factors. Furthermore, Clarivate acquired Proquest in 2021, a prominent global provider of software, data and analytics for academic, research and national institutions. Meanwhile, SpringerNature and Elsevier have been actively constructing comprehensive platforms to capture researchers’ data and develop new research analytics, aiming to enforce compliance with their established metrics and standards (see also our companion article [6]). These metrics and standards hold researchers and their employers captive within the confines of their platforms [31,32]. Initially resistant to OA, these same companies have now become the largest providers of OA by effectively monopolizing the market through highly profitable Article Processing Charges [33] and the new rhetoric of open by their definitions [34]. Consequently, many prominent society publishers find themselves trapped within this system, unable to break free.

The behaviour of scholarly societies with regard to journal publishing illustrates how some of them appear to have prioritized their own revenues over the interests of their members, scholarship at large and the public. While money is required to keep programmes running and initial quality concerns may have been understandable at the time, today, it appears anachronistic to risk the mission of the society citing financial concerns: there are numerous societies which thrive and prosper despite, no, because they embrace the opportunities provided by open digital infrastructure when the opportunity arises. It does not seem far-fetched to speculate that part of the motivation of scholars to use private social media platforms may be the focus of their societies on the financial, rather than the social aspects of their community.

## 7. The academic approach to the digital age

The hugely heterogeneous positions taken by scholarly societies with regard to digital technologies mirrors the approach by academia at large: some embrace digital technology, some oppose it, while the majority seems content hiding in a digital cave. Decentralized solutions require *cooperative* actions and shared interests, a common goal even, in a time when most scholarly institutions use ‘*competitive*’ to describe themselves and expect no less from their faculty and students. The documented actions of the scholarly societies help answer the question above as to why digital solutions in general and, hence, decentralized infrastructures for scholarly communication in particular, have taken decades to materialize. They raise the suspicion that, in contrast with the early 1990s when internet technology was implemented in scholarly institutions around the globe, there is currently no broad understanding, let alone a consensus, that actually implementing the digital technical developments since the 1990s would save tax funds, improve the work of faculty as well as the learning of students and benefit the society that funds public scholarship.

## 8. Realizing the ‘social’, from tweet to monograph

Historically, scholarly communication has always taken many forms: letters between individual scholars, meetings, journal articles or monographs. From the early days, the scholarly record is sketchy, later, only the journal and monograph portion is retained (as well as, for some fields, meeting papers). Scholarly discourse on Twitter formed a scholarly community there, #ScienceTwitter [3], and some of its members, particularly those of the Global South lacking access to many expensive resources, seem hesitant to leave, despite the mayhem. This separation between tweets, articles, monographs, etc. is largely historical. The categorization of tweets, articles, monographs and other forms of communication has largely been shaped by historical and social factors. The conventions surrounding scientific communication and what constitutes a publication have evolved over time, driven by institutional and disciplinary requirements, as well as advancements in technology [35]. It is crucial to recognize that the establishment of ‘peer-review’ as we know it today was primarily institutionalized during the Cold War era, partly in response to funders' demands for transparency and accountability in public expenditure [36]. Capitalizing on the need for accountability and independent validation of scientific research, for-profit publishers further legitimized a set of homogenized publishing standards that align with their workflow and profit generation goals. These standards also catered to the desire for standardized research outputs that could be easily quantified and measured [37]. However, the recent rise of preprint platforms, open peer-review models, experimentation by initiatives like eLife, the worldwide call for assessment reforms (e.g. Declaration on Research Assessment (DORA) and Coalition for Advancing Research Assessment (CoARA)), and the use of social media for scientific discourse and community-building demonstrate that scientists are no longer satisfied with the existing status quo. Journals may once have served as important sites for community-building but they have been turned into accounting and profit centres. Any organization where community-building, discourse and knowledge dissemination was a top priority and that had ‘social’ in the root of their name would probably have put the implementation of social technology at the top of their agenda at the latest when FriendFeed was bought by Facebook, probably much earlier. Even for those for whom it may not have been obvious back then, it is probably more clear now that communities are built online, journals become less and less relevant and discourse does not wait until the annual meeting. Perhaps these developments were accelerated by those societies that turned their journals from community-building venues into cash cows, driving scholars away in search for alternative ways to build and grow their communities?

We propose reclaiming ownership over the scholarly commons (see [38–46] for more detail), to be able to maintain the scholarly record from toot to monograph (and the code and data in-between). Analogous to other, non-digital areas of infrastructure, the infrastructure supporting the scholarly commons needs to come under the governance of the scholarly community. An example of an existing effort in this direction is the abovementioned CORE project based on the COAR Notify Initiative [47,48]. This project is developing and accelerating community adoption of a standard, interoperable and decentralized approach (using Linked Data Notifications) to link research outputs hosted in the distributed network of repositories with resources from external services, such as overlay-journals and open peer-review services. Our companion article [6] contains more detail on the larger scholarly infrastructure supporting the social technologies we are dealing with here (figure 1).

**Figure 2.**
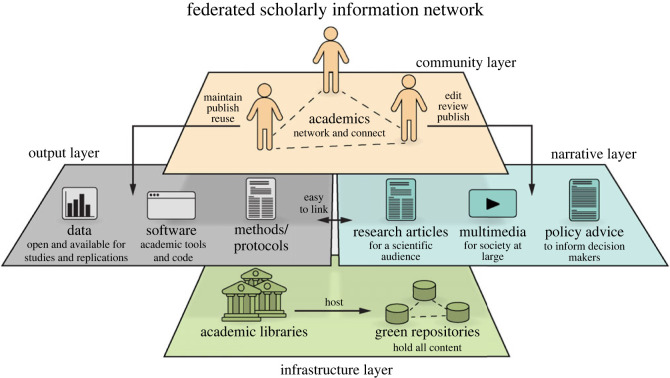
Concept for a federated scholarly information network. A federated network of institutional repositories constitutes the underlying infrastructure. Ideally, this infrastructure is designed redundantly, such that large fractions of nodes may go offline and the remaining nodes still provide 100% of the content. Users only directly interact with the output and narrative layers. The output layer contains all research objects, text, data and code. The narrative layer combines research objects in various forms, including research articles. The community layer encompasses the social technologies we are referring to in this article. See also our companion publication [6]. Modified from [49].

Internet and web standards showed the way. In academia, the worldwide repository network enabled by the Open Archives Initiative Protocol for Metadata Harvesting was the very foundation of decentralized infrastructure from the start [50], with CORE and Notify as a consequential extension. The Fediverse and Mastodon are only the latest instantiations of this concept. As non-profit organizations or charities, many societies have by-laws preventing corporate capture, similar to public research and teaching institutions. Scholarly societies are hence ideally placed not only to develop and implement all the necessary components, but also to ensure core scholarly values are reflected in this infrastructure and remain so over time (see also [51]). For instance, the server rules of their instances may be modelled after the code of conduct for their meetings. Scholarly societies ought to represent the scholarly community, rich and poor. As with earlier opportunities, the reactions of societies will probably differ: some will realize the opportunity because they are constantly seeking for new ways to contribute solutions and improvements, build communities around their fields and help support a public good, while others will be hesitant, wondering what is in it for them? Scholarly societies today face the choice of embracing the digital Mastodon or face the fate of the analogue Mastodon.

Some scholarly societies may have missed earlier opportunities, but now they are presented with their second chance. Now would be the perfect time for scholarly societies to start making good on the ‘social’ at the root of their names and amend mistakes of the past. Mastodon over Mammon: every scholarly society that values scholarship over revenue now has a golden opportunity to show their true colours—implement a Mastodon instance for anybody who identifies with the topic of the society, scholar or layperson. Each instance contributes a share to a common infrastructure where the scholarly community determines the rules and not a profit-driven individual. At a bare minimum, scholarly organizations should follow the example of the Washington Post and provide means for their members to get verified on Mastodon [52]. If we, the scholarly community, manage to create a truly public square that cannot be taken over by private interests, it may become a blueprint for how to bring the remaining scholarly record (text, data and code) into the Fediverse as well. The technical potential of the Fediverse exceeds functionalities such as Mastodon and offers solutions to merge existing repository and peer-review solutions with what we now call social technology: the difference between toots, journal articles and monographs is more socio-political than technical (see [14,15] and our companion publication [6] for where to take this concept).

During the peer-review process of this article, the Council of the EU has adopted conclusions on scholarly publishing that echo our proposal here [53]. On the same day, 10 major research organizations came out in support of the Council document [54]. We take this as a strong endorsement of the concepts outlined in this and our companion article [6].

## Data Availability

This article has no additional data.
